# 

**Published:** 1829

**Authors:** 


### CONTENTS OF NO. III. VOL. III.

### ESSAYS AND CASES.

ART. I.—Practical Essays on Medical Education and the Medical Profession.—BY THE EDITOR. - - - 317

ART. II.—Case of cartilaginous Structure of the Cervix Uteri in a pregnant woman, requiring an incision through the Os Uteri to render delivery practicable.—BY G. COTTON, M. D. of Marietta, President of the Medical Society of Ohio. - 341

ART. III.—Some account of the efficacy of tight bandages and oil of turpentine, applied to the extremities in certain stages of Autumnal Fever.—BY WILLIAM MCCLAY AWL, M. D. of Somerset, Ohio. - - - - - 342

ART. IV.—A Case of Organic disease of the stomach, with the appearances after death.—BY SAMUEL MARTIN, M. D. Col. Reg. Chir. Lond. Soc. of Morgan county, Ohio. - - 344

ART. V.—Some account of the Symptoms and Treatment of the Dengue, as it appeared and prevailed in the southern and central parts of Ohio, in the spring of 1829.—BY DR. JOHN COOK BENNET, of McConnellsville. - - - - 347

ART. VI.—Brief notices of the Measles, which prevailed at Circleville, O. in the spring of 1829.—BY DR. JOHN COOK BENNETT. - - - - - - - 351

ART. VII.—Remarks on the Physiology of the Eye.—BY JAMES C. FINLEY, M. D. of Cincinnati. - - - . 355

ART. VIII.—A case of Morbus Cœruleus, attended with infiltration of blood under the hairy scalp.—BY DR. WILLIAM M. VORIS, of West Union, Ohio, - - - - 361

ART. IX.—An Inquiry into the Pathology and Treatment of Yellow Fever, as it prevails at New Orleans at this time, August, 1829.—BY DR. LYMAN CRONKRITE. - - 367

### REVIEWS AND BIBLIOGRAPHICAL NOTICES.

ART. XI.—The People's Doctors.—New Guide to Health; or Botanic Family Physician. Containing a complete system of practice upon a plan entirely new, &c.—BY SAMUEL THOMSON.

A course of fifteen Lectures on Medical Botany, denominated Thomson's new Theory of Medical practice, &c. delivered in Cincinnati, Ohio.—BY SAMUEL ROBINSON.

Cases of cures performed by the use of Swaim's Panacea.—BY WM. SWAIM.

The Compleat English Physician, or Druggist Shop Opened.—BY WILLIAM SALMON, London, 1693. pp. 1208.

The Pulmist; or Introduction to the art of curing and preventing the Consumption or chronic Phthisis. A medical essay, including a new and better distinction of its causes, kinds, remedies, diets and other peculiarities. By Professor Rafinesque, Ph. D. and Pulmist, Professor, &c. - - - 393

### MISCELLANEOUS INTELLIGENCE.

I. Analectic.

1. Iodine at the Hospital of St. Louis. - - - - 421
2. Camphor vaporized, a Remedy for Chronic Gout and Rheumatism. - - - - - - - 421
3. The use of Tartar Emetic in Large Doses recommended first in England. - - - - - 423
4. Efficacy of sponging with Cold water in Measles. - 423
5. Subcarbonate of Iron in Tetanus. - - 425
6. Cheiloplastic (operation de) operation for New Lip. - 426
7. Treatment of Fistulæ. - - - - 426
8. Recto-Vaginal Fistula. - - - 427
9. Spontaneous Combustion of both hands. - - 427
10. Debility of the Rectum. - - - - 428
11. Preservative power of Belladonna against Scarlet Fever. 429
12. Solution of Camphor. - - - 430
13. Fœtus affected with Fungous Hæmatodes. - - 431
14. Acute Watery Effusion in the Brain occurring without Inflammation. - - - - 431
15. Tartar Emetic in Large Doses. - - - 433
16. Partial Palsey cured by Strychnia locally applied. - 434
17. Inflammation of the Sinuses of the Brain. - - 435
18. On the detection of Corrosive sublimate. By M. Orfila. 436
19. New Organic Alkalies discovered in Cinchona Bark. 438
20. Ophthalmia Neonatorum. - - - 439
21. Influence of the age of the Parents on the Sex of their offspring. - - - - - 441
22. Mode of Suspending the Secretion of Milk. - 442
23. Treatment of Imperforate Rectum. - - 442
24. Supposed Hernia of the Lung, in consequence of Tight Stays. 445

II. Analytical.

25. Treatment of Fractures of the Thigh Bone. - - 445
26. Small Pox in Pittsburgh and its vicinity. - . 447
27. Tubercular Consumption. Injurious effects of the antiphlogistic treatment. - - - - - - - 447
28. Cynanche Trachealis. - - - - - - 451
29. Entropion cured by pulling down the eyelid. - - 452
30. Chloride of Sodium in Cancer. - - - 453
31. Fœtal bones coloured with Madder, administered to a sow. 454
32. Rhus Glabrum, a remedy for Ptyalism. - - 454

20. Radical cure of a Hydrocele, by the introduction of a needle into the Tunica Vaginalis. . . . 126
21. Nux Vomica in Chronic Diarrhæa & Intestinal Hemorrhage. 127
22. Diagnostic Symptoms of Dislocation of the Head of the Femur into the Ischiatic Notch. By Dr. Buchanan. . 127
23. Traumatic Tetanus—Pathology of. . . . 129
24. Ointment for Glandular Swellings. . . . 130
25. Caustic Paste. . . . . . 130
26. Collyrium Siccum for the Opacity of the Cornea. . 131
27. The Effects of entire Abstinence from Solid and Liquid Food. 131
28. Of the Medicinal properties of the Leontodon Taraxacum. 134

II. Analytical.

29. Mania a potu. . . . . . . 135
30. Neuralgias. . . . . . . 138
31. Colica Pictonum. . . . . . 140
32. Flour a remedy for Burns and Scalds. . . . 141
33. Fractures of the os Femoris. . . . . 142
34. Test for Prussic Acid. . . . . . 143
35. Depletion a cure for Grangrena Senilis. . . 144
36. Angina Pectoris. Nervous System. . . 146
37. Metallic Ligatures in Surgery. . . . 148
38. Treatment of the Cutaneous disease produced by certain vegetable poisons. . . . . 148
39. The London University. . . . . 149
40. Fatal Illness and Dissection of Dr. Gall. . 151
41. Death of Professor Gorham. . . . 153
42. Death of a Centenarian. . . . 154

III. Original.

43. Small Pox. . . . . . 155
44. Fatal Effect from Vaccination. . . 156
45. Extensive cellular inflammation from Vaccination. . 158
46. Bath Chalybeate Spring. . . . 159
47. First District Medical Society of Ohio. . . 160

---

### *NO. II.*

### ESSAYS AND CASES.

Art. I.—An Account of the Autumnal Fever of Georgia, from observations made during four years, in the counties of Jones and Baldwin. By James C. Finley, M. D. now of Cincinnati. 165
II. An account of a fatal case of Acute Negro Consumption, with the appearances on dissection. By Dr. John Bennett, of Newport, Kentucky. , , , , 193
III. Memoranda of a supposed case of Ruptured Uterus consequent upon a partial adhesion of the Vaginal parieties. By Richard F. Barrett, M. D. of Greensburg, Kentucky. 196
IV. A case of Epilepsy suspended by a Burn on the sole of the foot. Communicated by Dr. H. E. Green, of Greenupsburgh, Kentucky. , , , , , 199
V. Notes on a case of Spina Bifida. By the Editor. , 200
VI. An account of a singular case of Fœtal Monstrosity. By Dr. John Cook Bennett, of M'Connelsville, Ohio. , 213
VII. Medical Jurisprudence. Sequel of the case of John Birdsell, with remarks on Feigned Insanity. By the Editor. 215

### REVIEWS AND BIBLIOGRAPHICAL NOTICES.

Art. VIII. Lænnec on diseases of the Chest. Philadelphia, 1823.

Collin on the Stethescope, Boston, 1829. , , , 222

### MISCELLANEOUS INTELLIGENCE.

### I. Analectic.

1. On the condition of the Blood and Blood-vessels in Inflammation. , , , , , , 261
2. On the Coagulation of the Blood. , , , 264
3. On the Therapeutic properties of Morphine. , , 264
4. On Stranguary from Cantharides, and its relief. , 267
5. On the tests by which Morphine may be made known in cases of Poisoning. , , , , , 268
6. On the Influence of Mercury on the functions of the Uterus. 269
7. Cataract and Glaucoma. , , , 270
8. On small doses of oil of Turpentine for the cure of sciatica and other neuralgiæ. , , , , 271
9. On the form of the face and forehead; with a theory of their formation in different ages and nations. , , 272
10. Pathology of Muscæ Volitantes. , , , 274
11. Tartar on the Teeth. , , , , 275
12. New mode of administering Quinine. , , 576
13. On the supposed Existence of active Molecules in Mineral substances. , , , , , 276
14. On the Physiological Effects of Oxygen Gas, upon the Animal System. , , , , , 379
15. Silver found in the viscera of a person who had used the Nitrate of Silver. , , , , 280
16. Rhubarb plant. , , , , 280

17. On the Treatment of Intermittent Fevers by the Sulphate of Quinine, applied to a denuded surface. , , 280
18. Ergot of Rye in Chronic Uterine Discharges. , 281
19. Talicotian Operation performed with success. , , 282
20. Fungus Tumours from the Gum and Alveoli. , 282
21. Small Pox. , , , , , 283
22. Mercury detected in Swaim's Panacea, by Chemical analysis. 285
23. Some account of the Siamese boys, lately brought to Boston. 286
24. Extract from another account of the same. , , 288
25. A case of twins united by the Umbilicus. By JOSHUA MARTIN, M. D. of Xenia, Ohio. , , , 290
26. General Phlebitis. , , , , 291
27. Varieties of Hydrocele—Modifications of the usual operations. , , , , , 292
28. On the reduction of Herniæ. By M. DUPUYTREN. , 293
29. Supposed Artificial Diamonds. , , , 295

### II. ANALYTICAL AND ORIGINAL.

30. Wounds of the Heart. By Professor COXE. , , 295
31. Respiration of Children in Reversed Presentations. By Professor BIGELOW. , , , , 299
32. Aneurism. Tying the artery on the distal side. Mr. WALDROP. 301
33. Oil of Turpentine in increased Herniæ. By Prof. SEWALL. 303
34. Dengue. By Dr. DANIELL. , , , , 304
35. Modus operandi of poisons. , , , , 305
36. Morbid appearances after death, from Colica Pictonum. By E. J. COXE, M. D. , , , , , 312
37. Silliman's Journal. , , , , , 313
38. To our Readers. , , , , , 314

---

### *NO. III.*

### ESSAYS AND CASES.

ART. I.—Practical Essays on Medical Education and the Medical Profession.—BY THE EDITOR. . . . 317
II.—Case of cartilaginous Structure of the Cervix Uteri in a pregnant woman, requiring an incision through the Os Uteri to render delivery practicable.—BY G. COTTON, M. D. of Marietta, President of the Medical Society of Ohio. . . 341
III.—Some account of the efficacy of tight bandages and oil of turpentine, applied to the extremities in certain stages of Autumnal Fever.--BY WILLIAM MCCLAY AWL, M. D. of Somerset, Ohio. . . . . 342
IV.—A case of Organic disease of the stomach, with the appearances after death.—By Samuel Martin, M. D. Col· Reg. Chir. Lond. Soc. of Morgan County, Ohio. . 344
V.—Some account of the symptoms and Treatment of the Dengue as it appeared and prevailed in the southern and central parts of Ohio, in the spring of 1829.—By Dr. John Cook Bennett, of McConnellsville. . . . . 347
VI.—Brief notices of the measles, which prevailed at Circleville, O. in the spring of 1829.—By Dr. John Cook Bennett. 351
VII.—Remarks on the Physiology of the Eye.—By James C. Finley, M. D. of Cincinnati. . . . 355
VIII.—A case of Morbus Cœruleus, attended with infiltration of blood under the hairy scalp.—By Dr. William M. Voris, of West Union, Ohio. . . . . 361
IX.—An Inquiry into the Pathology and Treatment of Yellow Fever, as it prevails at New Orleans at this time, August, 1829.—By Dr. Lyman Conkrite. . . 367

REVIEWS AND BIBLIOGRAPHICAL NOTICES.

X.—The People's Doctors---New Guide to Health; or Botanic Family Physician. Containing a complete system of Practice upon a plan entirely new, &c.—By Samuel Thomson.
A Course of fifteen Lectures on Medical Botany, denominated Thomson's new Theory of Medical practice, &c. delivered in Cincinnati, Ohio.—By Samuel Robinson.
Cases of cures performed by the use of Swaim's Panacea.—By Wm. Swaim.
The Compleat English Physician, or Druggist Shop Opened.—By William Salmon, London, 1693. pp. 1208.
The Pulmist; or Introduction to the art of curing and preventing the Consumption or chronic Phthisis. A medical essay including a new and better distinction of its causes, kinds, remedies, diets, and other peculiarities. By Professor Rafinesque, Ph. D. and Pulmist, Professor, &c. 393

MISCELLANEOUS INTELLIGNCE.

I. Analectic.

1. Iodine at the Hospital of St. Louis. . . 421
2. Camphor vaporized, a Remedy for Chronic Gout and Rheumatism. . . . . 421
3. The use of Tartar Emetic in Large Doses recommended first in England. . . . . 423
4. Efficacy of sponging with cold water in Measles. . 423
5. Subcarbonate of Iron in Tetanus. . 425
6. Cheiloplastic (operation de) operation for New Lip. 426
7 Treatment of Fistulæ. . . . 426

8. Recto-Vaginal Fistulæ. . . . 427
9. Spontaneous combustion of both hands. . . 427
10. Debility of the Rectum. . . 428
11. Preservative power of Belladonna against Scarlet Fever. 429
12. Solution of Camphor. , , , 430
13. Fœtus affected with Fungous Hæmatodes. , 431
14. Acute Watery Effusion in the Brain occurring without Inflammation. , , , , 431
15. Tartar Emetic in Large Doses. , , , 433
16. Partial Palsey cured by Strychnia locally applied. , 434
17. Inflammation of the Sinuses of the Brain. , , 435
18. On the detection of Corrosive Sublimate. By M. Orfila. 436
19. New Organic Alkalies discovered in Cinchona Bark. 438
20. Ophthalmia Neonatorum. , , , , 439
21. Influence of the age of the Parents on the Sex of their offspring. , , , , , 441
22. Mode of Suspending the Secretion of Milk. , , 442
23. Treatment of Imperforate Rectum, , , , 442
24. Supposed Hernia of the lung, in consequence of Tight Stays, 445

II. Analytical.

25. Treatment of Fractures of the Thigh Bone, , 445
26. Small Pox in Pittsburgh and its vicinity, , , 447
27. Tubercular Consumption. Injurious effects of the antiphlogistic treatment, , , , , , 447
28. Cynanche Trachealis, , , , 451
29. Entropion cured by pulling down the eyelid, , , 452
30. Chloride of Sodium in Cancer, , ; , 453
31. Fœtal bones colored with madder, administered to a sow, 454
32. Rhus Glabrum, for Ptyalism, , , , 454

---

### *NO. IV.*

### ESSAYS AND CASES.

Art. I.—Facts and observations on the Milk Sickness, or Sick Stomach of Tennessee, in a letter to Dr. Samuel Brown, from Dr. Alexander M'Call. . . . . 465
II.—History of a successful case of Cæsarian operation, By Dr. John L. Richmond, of Newtown, Ohio. . . 485
III.—A case illustrative of the effects of the Colchicum Autumnale, by Dr. R. J. Buckner, of Brown Co. Ohio. . 489
IV.—Observations on the Ætiology of Bilious Fever by Dr. John Cook Bennett, of M'Connellsvile, Ohio, · 494

V.—Cases illustrating the effects of the Roller Bandage, by Joseph K. Sparks, M. D. of Cincinnati. . . . 497

### REVIEWS AND BIBLIOGRAPHICAL NOTICES.

VI.—Malaria; an essay on the production and propagation of this poison, and on the nature and localities of the places by which it is produced, with an enumeration of the diseases caused by it, &c. By John Macculloch M. D. F. R. S. &c. &c.; by a Correspondent. . . . 500

VII.— A Treatise on the Scrofulous disease, by C. G. Hufeland, Physician to the King of Prussia, &c. Translated from the French of M. Bosquet, by Charles D. Meigs, M. D. 545

### MISCELLANEOUS INTELLIGENCE.

### I. Analectic.

1. Mr. Battley's liquor Cinchonæ Cordifoliæ. . 561
2. Peculiar Odorous Principle in the blood of man distinguishing it from that of other animals. . . . 566
3. Observations on the use of the Ergot Rye. . 568
4. Nitrate of Silver. . . . . 569
5. Human monster with two heads. . . 569
6. Experiments proving that the nervous Tissue possesses the property of developing the Galvanic fluid. . 572
7. Hydrophobia. . . . . 574
8. On the effects of Camphor in a healthy individual. 575
9. Treatment of persons poisoned by Opium. . 576
10. Treatment of persons poisoned with Hydrocyanic acid, 576
11. Mode of stopping Hæmorrhage by twisting the mouth of the vessels. . . . . . 577
12. New mode of employing the Nitrate of Silver as a test for arsenic. . . . . . . 578
13. Power of the sulphate of Quinine in accelerating Mercurial Action. . . . . . 579
14. Tetanus cured by the acetate of Morphia applied externally. 580
15. Dr. Wedemeger, on the circulation. . . 581
16. Treatment of Gonorrheal Ophthalmia. . . 583
17. Treatment of Ophthalmia Neontorum. . 584
18. Iodine in Scrofula. . . . . 585
19. On the action of the Arteries in the Arterial Circulation. 585

### II. Analytical.

20. Anticardal operation for aneurism. . . . 587
21. Pyroligneous acid in ulcers and gangrene. . 589
22. Tartar Emetic ointment in chorea. . . . 590

23. Sloughing of twenty-six inches of intestine. . 590
24. Treatment of Pulmonary consumption. . . 590
25. Stethoscopic diagnosis of pregnancy. . . 594
26. Medical Jurisprudence, Mania a potu. . . 598
27. Hospital Surgeons of Paris. . . . . 603

III. ORIGINAL.

28. Notes of a case of nervous irritation during pregnancy with separation of the symphysis Pulbis. . . 604
29. Obituary notice of Professor Brown. . . 606
30. Jefferson Medical College. . . . . 608
31. Jefferson Medical College commencement. . . 610
32. To subscribers and the profession generally. . 610

